# *Cryptosporidium* genotypes in children and calves living at the wildlife or livestock interface of the Kruger National Park, South Africa

**DOI:** 10.4102/ojvr.v83i1.1024

**Published:** 2016-05-20

**Authors:** Nada Abu Samra, Ferran Jori, Simone M. Cacciò, John Frean, Bhavani Poonsamy, Peter N. Thompson

**Affiliations:** 1 Department of Production Animal Studies, University of Pretoria, South Africa; 2 French Research Institute for Agricultural Development (CIRAD), Integrated Animal Risk Management Unit (UPR AGIRs), Campus International de Baillarguet, France; 3 Department of Animal Science and Production, Botswana College of Agriculture, Botswana; 4 Department of Infectious, Parasitic and Immunomediated Diseases, Istituto Superiore di Sanità, Italy; 5 Centre for Opportunistic, Tropical and Hospital Infections, National Institute for Communicable Diseases, South Africa; 6 Research Institute for Malaria, University of the Witwatersrand, South Africa

## Abstract

*Cryptosporidium* infection is one of the most common causes of parasitic diarrhoea worldwide in cattle and humans. In developing countries, human cryptosporidiosis is most prevalent during early childhood and links between zoonotic infection and animal related activities have been demonstrated. This study investigated the prevalence and species/genotype distribution of *Cryptosporidium* among children (< 5 years) and calves (< 6 months) living in a rural farming area adjacent to the Kruger National Park in South Africa, where interactions between humans and wild and domestic animals are known to occur. *Cryptosporidium* oocysts were detected in 8/143 stool samples of children recruited within the hospital system (5.6%; 95% CI 2.4%, 10.7%) and in 2/352 faecal samples of calves (0.6%; 95% CI 0.1%, 2.0%) using the modified Ziehl–Neelsen (MZN) staining technique. Microscopy positive samples from children were further analysed by PCR targeting the 18S rRNA gene and identified as *Cryptosporidium hominis* (3/4) and *Cryptosporidium meleagridis* (1/4). Regardless of the microscopy outcome, randomly selected samples (*n* = 36) from calves 0–4 months of age were amplified and sequenced at the 18S rRNA gene using nested PCR. Two calves tested positive (5.6%; 95% CI 1.7%, 18.7%), and revealed the presence of *Cryptosporidium parvum* and *Cryptosporidium bovis*. The detection of only two zoonotic species (*C. parvum* in one calf and *C. meleagridis* in one child) suggests that zoonotic cryptosporidiosis is not currently widespread in our study area; however, the potential exists for amplification of transmission in an immunocompromised population.

## Introduction

*Cryptosporidium* spp. are Apicomplexan parasites that infect a wide range of vertebrates, including humans. Cryptosporidiosis is associated with mild to severe diarrhoea that is typically self-limiting in immunocompetent hosts, but can become chronic and life-threatening in immunocompromised and very young individuals (Ramirez, Ward & Sreevatsan 2004).

Humans are most commonly infected by *Cryptosporidium parvum*, which primarily infects cattle and may be of either zoonotic or anthroponotic origin, and by *Cryptosporidium hominis*, which primarily infects humans. Other species of *Cryptosporidium*, including *Cryptosporidium meleagridis, Cryptosporidium felis,* and *Cryptosporidium canis*, are responsible for a smaller number of infections in humans and have mainly been reported in immunosuppressed patients (Xiao & Feng 2008). More recently, *C. meleagridis* has been recognised as an emerging human pathogen (Putignani & Menichella 2010; Xiao 2010) and was responsible for up to 10% of all infections in developing countries (Gatei *et al*. 2006; Xiao 2010).

The zoonotic importance of *Cryptosporidium* spp. has been recognised for some time (Xiao & Feng 2008); however, the role of wild and domestic animals in the epidemiology of human cryptosporidiosis, particularly in developing countries, is not completely understood. *C. parvum* is the only species of zoonotic importance identified in cattle and several outbreaks of *C. parvum* in people have been associated with infected calves in the United States and the United Kingdom (Gait *et al*. 2008; Kiang *et al*. 2006; Smith *et al*. 2004). In Africa, specifically in Egypt and Ethiopia, contact with cattle has been identified as a significant risk factor for the zoonotic transmission of *C. parvum* in children and HIV and/or AIDS patients (Adamu *et al*. 2014; Helmy *et al*. 2013).

In South Africa, cryptosporidiosis has been reported in humans and livestock in various locations. In diarrhoeic children a prevalence of 24.8% was found in a hospital in Durban (Leav *et al*. 2002), and more recently an overall prevalence of 12.2% was reported in hospitalised diarrhoeic children from four different provinces, with a prevalence of 8.6% in one of the four hospitals located in close proximity to our study area (Abu Samra *et al*. 2013a). The predominant species found in both studies was *C. hominis*, with five different GP60 subtype families (Ia, Ib, Id, Ie, and If), followed by anthroponotic forms of *C. parvum* (IIc, IIe, and IIb); this supported previous findings that suggested that cryptosporidiosis in developing countries is predominantly of anthroponotic origin (Mor & Tzipori 2008; Xiao 2010). In cattle, data on cryptosporidiosis are scarcer: a prevalence of 33.6% (36/107) with *Cryptosporidium* spp. has been reported in adult cattle from the southern Free State Province, South Africa (Bakheit *et al*. 2008) and, more recently, a study conducted in a different area adjacent to the Kruger National Park (KNP) reported a prevalence of 8% (4/51) in weaned calves, with *Cryptosporidium bovis* and *Cryptosporidium andersoni* as circulating genotypes (Abu Samra *et al*. 2013b).

Because no simultaneous investigation on *Cryptosporidium* spp. in young children and animals living at the wildlife/livestock/human interface has been undertaken to date, the objective of this study was to estimate the prevalence and study the genetic diversity of *Cryptosporidium* spp. and to identify potential risk factors associated with cryptosporidiosis in young children and calves in an area of the KNP wildlife/livestock/human interface.

## Materials and methods

### Study site

**FIGURE 1:**
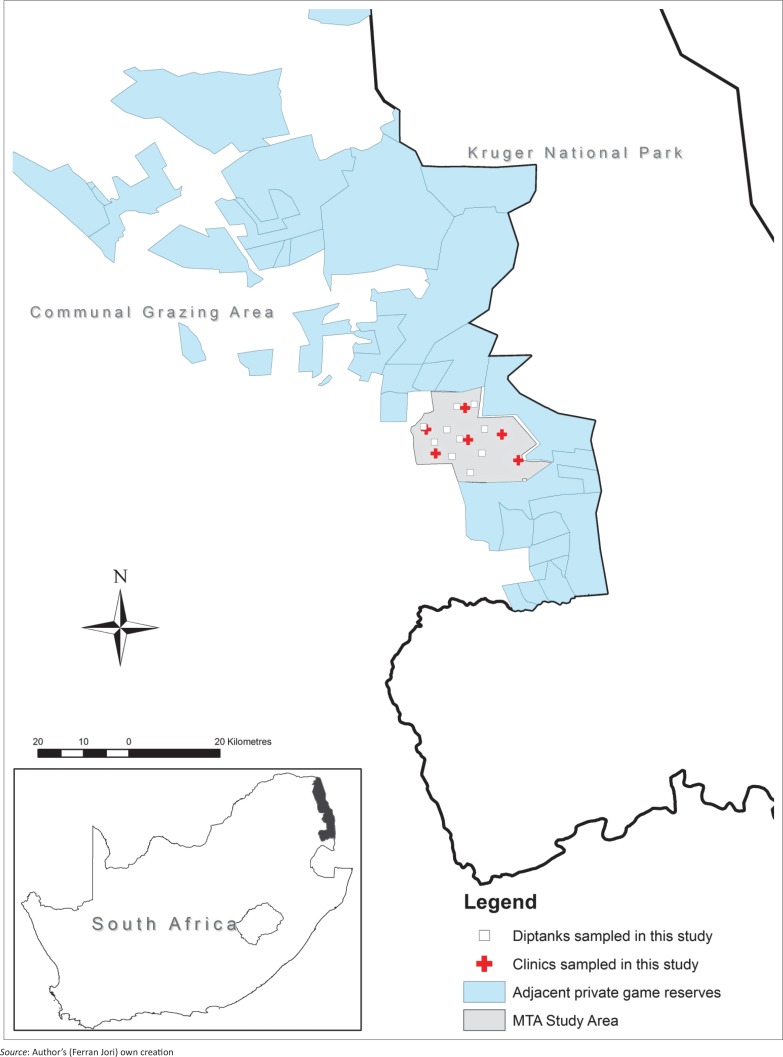
Study area for the location of clinics and dip tanks where sampling took place.

The study took place in a communal farming area, the Mnisi Traditional Authority (MTA), situated in a rural environment in the north-eastern Lowveld area of Mpumalanga Province, South Africa, adjacent to private protected wildlife areas continuous with the KNP (Figure 1). In this area, livestock production is a prevalent activity among rural communities, and contact between cattle and wildlife occurs (Abu Samra *et al*. 2011, 2013a; Brahmbhatt *et al*. 2009). The local presence of *Cryptosporidium* spp. in cattle and wildlife in the area had previously been reported (Abu Samra *et al*. 2013a).

In this community, subsistence farmers rely mostly on livestock farming, the most common livestock being the indigenous Nguni cattle, used for milk and meat production. Cattle dip tanks have been established throughout the region, and every cattle herd is taken for dipping and inspection once a week to control tick-borne diseases (Simela 2012). The MTA community hosts a population of more than 40 000 people and eight human health centres are located within this community. It is estimated that approximately 34% of the human population of MTA is infected with human immunodeficiency virus (HIV) (Moshabela *et al*. 2011).

## Ethical consideration

This study was approved by the Animal Use and Care Committee of the University of Pretoria (Protocol V014/08) for animal sampling, and by the Research Ethics Committee of the University of Pretoria for human sampling (Protocol no. 5/2011). Before initiating the work, all stakeholders were contacted in order to seek their permission. Animal health technicians and a local field assistant assisted in informing all cattle owners of the purpose of this study and that participation was voluntary. Nurses at participating health care centres informed their patients of the purpose of the study and if parents agreed to their child’s participation, a consent form was signed. Finally, stool samples were collected with parental consent and on a voluntary basis in collaboration with nurses working at each of the clinics.

### Sample collection

From March to June 2012, during autumn, at the end of the rainy season and the start of the dry season, stool samples from 143 children < 5 years old were collected by convenience sampling from six rural clinics in the study area, without specific consideration for gender or reported symptoms. The parents of each participant were asked to complete a brief questionnaire to gather information on their child’s age, contacts with domestic animals, source of drinking water, whether water was boiled before drinking, and history of diarrhoea since birth.

Simultaneously, 352 faecal samples were collected from pre-weaned calves (< 6 months of age) at 11 dip tanks in the study area. A sample size was calculated to estimate a 5% prevalence with 2.5% allowable error, using the formula *n* = 1.96^2^*PQ*/*L*^2^ (Thrusfield 2007), where *P* is the estimated prevalence (0.05), *Q* = 1–*P* and *L* is the absolute allowable error (0.025), giving a required minimum sample size of 292. Faecal samples were collected directly from the rectum of calves and each dip tank was visited only once to avoid repeated sampling of individual calves. A brief questionnaire was completed by interviewing the herdsman accompanying each sampled animal. Closed questions (dichotomous and multiple choice) for ascertaining age, herd size, and source of drinking water were used.

All faecal samples from children and calves were transported on ice to the National Institute for Communicable Diseases (NICD) in Johannesburg, where they were preserved in 2.5% potassium dichromate and stored at 4 °C until processed.

### Laboratory analysis

At the NICD faecal samples from calves and children were concentrated by the formalin-ethyl acetate sedimentation method and thin smears were stained using the modified Ziehl–Neelsen (MZN) technique (Garcia 2001). The stained slides were examined by light microscopy with immersion oil using 50x and 100x objectives to detect *Cryptosporidium* spp. oocysts. Because of limited resources only MZN-positive faecal samples of children (*n* = 8) were sent to the European Union Reference Laboratory for Parasites at the Instituto Superiore di Sanità in Rome, Italy, for molecular analysis. Of the calf samples, only those from animals within the 0–4 month age group were further analysed; 36 samples of this age category were randomly selected for molecular analysis, irrespective of their MZN outcome.

### DNA extraction

Prior to DNA extraction, *Cryptosporidium* oocysts were purified from the faeces by caesium chloride gradient. Purified oocysts were suspended in 25 µL of buffer (10 mM Tris pH 8.3, 50 mM KCl containing 0.5% w/v Tween 20). After freeze-thawing (15 cycles), samples were heated for 15 min at 100 °C and then centrifuged for 2 min at 13 000 rpm to remove particulate matter. Finally, 2 µL – 5 µL of supernatant was used for PCR amplification (Bonnin *et al*. 1996).

### Amplification and genotyping at the 18S rRNA locus

The extracted DNA of samples from children (*n* = 8) and calves (*n* = 36) were amplified at the 18S rRNA gene using a two-step nested PCR assay as previously described. In short, a primary PCR product of 760 bp was amplified and for the secondary PCR a fragment of about 590 bp was amplified (Ryan *et al*. 2003). The amplified 18S rRNA gene fragment was analysed by electrophoresis on 2% agarose gels, stained with ethidium bromide and visualised under a UV transilluminator. The secondary PCR products were purified and sequenced on both strands. Sequences were assembled using the software SeqMan version 7.1 (DNASTAR, Inc.) and compared with those available in GenBank using BLAST software (Pruitt, Tatusova & Maglott 2005).

### Cryptosporidium amplification and sequencing of the GP60 gene

DNA of all samples identified as *C. hominis* were then amplified at the GP60 gene, using external primers (Strong, Gut & Nelson 2000) and internal primers (Drumo *et al*. 2012). PCR products were purified using spin columns and sequenced on both strands. Sequences were assembled using SeqMan version 7.1 (DNASTAR, Inc.). A BLAST search against the GenBank database was used to identify *C. hominis* GP60 subtypes.

### Statistical analysis

Data were entered into a spreadsheet and then analysed using Epi-Info (Version 3.3.2., CDC Atlanta, 2005) and Stata 12.1 (StataCorp, College Station, TX, USA). Prevalences were calculated with 95% exact binominal confidence limits. The bivariable association of each variable (potential risk factor and history of diarrhoea) with *Cryptosporidium* infection was assessed separately for calves and for children using Fisher’s exact test. In addition, all potential risk factors were entered into multivariable exact logistic regression models to adjust for confounding. Statistical significance was assessed at *P* < 0.05.

## Results

### Children

From faecal samples of 143 children, eight were found positive by the MZN staining method and were confirmed by PCR. Therefore, the prevalence of *Cryptosporidium* infection in children was estimated to be 5.6% (95% CI 2.4%, 10.7%). At least one positive sample was detected in children in five out of the six clinics surveyed.

Four of the eight 18S rRNA PCR-positive samples were successfully sequenced and *C. hominis* (3/4) and *C. meleagridis* (1/4) were identified. Sequence analysis of the GP60 gene was done for the *C. hominis* isolates and two subtype families were identified, namely Ib (IbA12G3R2 and IbA10G2) and Ie (IeA11G3T3). Sequences were deposited in GenBank (Submission ID 1871551).

Bivariable associations were assessed with potential risk factors from 141 questionnaires (two interviews were not completed) (Table 1). Details on diarrhoeic episodes were not systematically reported and therefore only history of diarrhoea was considered during the analysis. The univariable associations of the five potential risk factors with the presence of *Cryptosporidium* in children were not significant; likewise, the exact logistic regression model was unable to detect significant associations between any of the factors and *Cryptosporidium* infection.

**TABLE 1:** Potential risk factors associated with Cryptosporidium infection in 143 children < 5 years old: Results of a multivariable exact logistic regression model.

Variable	Level	Odds ratio	95% CI (OR)	*P*
Age	0–2 months	1†	-	-
	<6 months	0.62	0.01, 55.7	1.000
	6–12 months	0.89	0.07, 51.2	1.000
	>12 months	0.34	0.01, 26.7	0.880
Contact with cattle	No	1†	-	-
	Yes	0.87	0.07, 7.10	1.000
Contact with other animals (cat, dog, goat)	No	1†	-	-
	Yes	1.31	0.17, 15.5	1.000
Source of drinking water	other	1†	-	-
	tap	0.08	0.002, ∞	1.000
Boiling water before drinking	No	1†	-	-
	Yes	1.09	0.01, 20.3	1.000

† Reference level.

### Calves

Only 2/352 calf samples (0.6%; 95% CI 0.1%, 2.0%) were positive by MZN and of the 36 calves < 4 months of age randomly selected for 18S rRNA nested PCR (which did not include the two MZN-positive samples) two were positive, giving an estimated prevalence of 5.6% (95% CI 1.7%, 18.7%). None of the univariable associations of the three potential risk factors with the presence of *Cryptosporidium* in calves were significant and the exact logistic regression model was unable to detect any significant associations.

Sequencing of the 18S rRNA gene PCR product revealed the presence of *C. parvum* (100% homology to many *C. parvum* sequences, e.g. KJ719487) and *C. bovis* (100% homology to many *C. bovis* sequences, e.g. KJ531689) in the two positive samples.

## Discussion

The prevalence of *Cryptosporidium* spp. infection detected in children and calves in this study is lower compared to previous studies conducted in southern Africa (Samie *et al*. 2006; Siwila *et al*. 2011). Cryptosporidiosis is recognised as an important disease in children and immunosuppressed adults in sub-Saharan Africa (Mor & Tzipori 2008), but with a widely varying prevalence. In Uganda, for example, a prevalence of 25% was detected among diarrhoeic children and of 8.5% among non-diarrhoeic children (Tumwine *et al*. 2003). *Cryptosporidium* was identified in Tanzania in 13 and 9% of children with acute and chronic diarrhoea respectively, but no oocysts were detected in children without diarrhoea (Cegielski *et al*. 1999). In South Africa, cryptosporidiosis has been reported by different studies at prevalence ranging between 12% and 25% of hospitalised diarrhoeic children, including a prevalence of 8.6% (11/128) in Agincourt Hospital, located within 20 km from our study area (Abu Samra *et al*. 2013b; Leav *et al*. 2002).

The prevalence of *Cryptosporidium* spp. in calf samples was low compared to studies targeting calves of a similar age group elsewhere in Africa. In Nigeria, for instance, a prevalence of 16% was reported in native cattle less than 1 year of age (Maikai *et al*. 2011). In Zambia, 48% of dairy calves and 6.3% of traditionally-raised calves, all less than 3 months of age and within the same study area, were found to be infected with *Cryptosporidium*, suggesting that native breeds of cattle from traditional husbandry systems may be more resistant to *Cryptosporidium* infection compared to intensively raised cattle (Geurden *et al*. 2006).

The lower prevalence observed in children and calves in this study may be because our sampling did not specifically target diarrhoeic individuals, who are known to shed a high number of *Cryptosporidium* oocysts in their faeces (DuPont *et al*. 1995). The diagnostic method used for the detection of *Cryptosporidium* oocysts (MZN stain) has a reduced sensitivity for detecting *Cryptosporidium* in low-oocyst-shedding individuals. Indeed, other studies using more sensitive molecular techniques have shown considerably higher detection rates. In Uganda for instance, PCR was 32% more sensitive in detecting *Cryptosporidium* oocysts than was MZN (Tumwine *et al*. 2003). In Egypt, human samples were analysed by non-molecular and PCR techniques and prevalences of 6.7% and 49% were detected, respectively (Helmy *et al*. 2013).

Another aspect that could have influenced our results is the period of sampling, which spanned the end of the rainy season and the start of the dry season. Seasonal variation of cryptosporidiosis in human and animals has been reported in a number of studies in southern and eastern Africa, such as in Malawi (Morse *et al*. 2007), Uganda (Tumwine *et al*. 2003), and Zambia (Nchito, Kelly & Sianongo 1998), with the highest prevalence recorded during the rainy season.

Results of the PCR and sequencing analysis of the 18S rDNA gene identified *C. hominis* as the dominant species in children, which is in agreement with previous findings elsewhere in Africa (Akiyoshi *et al*. 2006; Gatei *et al*. 2007; Morse *et al*. 2007). Four *C. hominis* subtype families are most commonly observed in humans in developing countries: Ia, Ib, Id, and Ie (Xiao 2010). The *C. hominis* subtype family most frequently detected in this study was Ib (2/3), followed by Ie (1/3). Within the *C. hominis* subtype family Ib, IbA10G2 has been previously described in South Africa, Peru, and India (Cama *et al*. 2008; Gatei *et al*. 2007; Leav *et al*. 2002) and was identical to the one identified in this study. Although the number of successfully sequenced samples (4/8) was too small to draw any conclusions, a recent study from a hospital located in close proximity to our study area, where a predominance of anthroponotic species (100%) was found, is consistent with our findings (Abu Samra *et al*. 2013b).

Regarding zoonotic species, our study detected the circulation of *C. meleagridis* in one child and *C. parvum* in one calf. *Cryptosporidium meleagridis*, reported in a wide range of wild and domestic mammals, including rodents, cattle, and humans (Caccio 2005; Cama *et al*. 2008; Qi *et al*. 2011; Xiao 2010; Xiao *et al*. 2002), is regarded as an emerging human pathogen (Putignani & Menichella 2010) and has been reported before in diarrhoeic children in South Africa (Abu Samra *et al*. 2013b). Likewise, *C. parvum* is known to be an important cause of zoonotic cryptosporidiosis worldwide (Gait *et al*. 2008; Hunter & Thompson 2005; Kiang *et al*. 2006; Smith *et al*. 2004); however, the low prevalence of *C. meleagridis* and *C. parvum* detected during this study suggests that zoonotic cryptosporidiosis is currently not widespread in our study population.

We were not able to detect any association between animal contact and *Cryptosporidium* infection in children in our study; the low prevalence of cryptosporidiosis reduced the power of the statistical analysis to identify risk factors. The source of drinking water as a potential risk factor for *Cryptosporidium* infection in children and calves was one of the variables investigated during this study; however, pipe-borne water was used by the large majority (99%) of households interviewed, which suggests that water sources were generally not shared with animals, therefore reducing the probability of zoonotic infection in our studied community. Water-borne pathogens, such as *Cryptosporidium* spp. in rural communities in developing countries, can be amplified by seasonal water flows and flooding, which facilitate the common use of contaminated water sources between humans and animals (Alexander *et al*. 2013; Fenwick 2006). In a similar study assessing epidemiological and molecular interactions between humans, wildlife, and livestock in Uganda, fetching water from an open water source was suspected to increase the probability of cryptosporidiosis in humans (Salyer *et al*. 2012).

The fact that zoonotic cryptosporidiosis was detected in only one child and one calf suggests that zoonotic transmission may not be important in our study area. However, considering that the prevalence of HIV among rural communities in South Africa is among the highest in the world and was estimated to be 34% in our study area (Moshabela *et al*. 2011), the potential impact of the circulation of zoonotic strains in our study should be investigated further.

## Conclusion

Considering that children and calves are good indicators of cryptosporidiosis in their respective populations, zoonotic cryptosporidiosis appears to occur at low levels in our studied community at the interface of the KNP. However, the detection of some zoonotic strains does not allow us to exclude a potential zoonotic risk and impact in an immunocompromised human population. Therefore, further studies should be undertaken, considering different spatio-temporal settings, using molecular diagnostic tools, and simultaneously assessing the prevalence of oocysts in domestic and wild animals, children, and water.
